# Interprofessional collaboration milestones: advocating for common assessment criteria in graduate medical education

**DOI:** 10.1186/s12909-015-0432-0

**Published:** 2015-09-14

**Authors:** Majken T. Wingo, Rachel DA Havyer, Nneka I. Comfere, Darlene R. Nelson, Darcy A. Reed

**Affiliations:** 1 Division of Primary Care Internal Medicine, Department of Medicine, Mayo Clinic, Rochester, MN USA; 2 Departments of Dermatology and Laboratory Medicine & Pathology, Mayo Clinic, Rochester, MN USA; 3 Division of Pulmonary and Critical Care Medicine, Department of Medicine, Mayo Clinic, Rochester, MN USA

## Abstract

**Background:**

Milestone-based assessments of resident physicians inform critical decisions regarding resident competence and advancement. Thus, it is essential that milestone evaluations are based upon strong validity evidence and that consistent evaluation criteria are used across residency programs. A common approach to assessment of interprofessional collaboration milestones is particularly important since standardized measures of individual resident competence in interprofessional collaboration have not been established.

**Discussion:**

We propose that assessments of interprofessional collaboration in graduate medical education meet common criteria, namely, these assessments should: 1) measure competency of an individual resident, 2) occur in the context of an interprofessional team, 3) be ascertained via direct observation of the resident, 4) be performed in a real-world clinical practice setting (such as a hospital ward, outpatient clinic, or operating room). We present the evidence-based rationale for these criteria and cite examples of published assessment instruments that fulfill one or more of the criteria, however further research is needed to ensure fidelity of assessments.

**Summary:**

The proposed criteria may assist residency educators as they endeavor to provide robust and consistent assessments of interprofessional collaboration milestones.

## Background

In 2013, the Accreditation Council for Graduate Medical Education introduced the Educational Milestone framework for assessment of residents in the Next Accreditation System [1]. Milestones are a sequence of developmentally-based behaviors that residents are expected to demonstrate throughout training [2]. Milestones are used both to assess the competence of individual residents, as well as to support the accreditation of residency programs [1]. Since milestones may potentially inform high-stakes decisions regarding resident advancement and program accreditation, it is critical that milestone assessments are based on strong validity evidence and are performed according to uniform criteria across residency programs.

Each specialty has published a list of milestones, which implies consensus within specialties regarding *what* must be assessed. There is no consensus, however, regarding *how* milestone assessments must be performed. Although there is broad acknowledgement that all assessments should be supported by validity evidence according to established frameworks [3, 4], beyond this, it is left up to individual program directors and teaching faculty to decide how to assess each milestone. In the absence of common criteria for assessment, important decisions may be made using subjective and variable measures.

Lack of a common approach to assessment is particularly problematic for milestones related to interprofessional collaboration and teamwork because, unlike medical knowledge and clinical skills, nationally standardized measures of individual resident competence in interprofessional collaboration have not been established [5]. Although numerous teamwork instruments exist, the most extensively studied instruments with the strongest validity evidence, such as the Safety Attitudes Questionnaire [6–10] and the Team Climate Inventory [11–13], assess clinical teams *as a whole* rather than individual residents on a team. Yet milestones require assessments of individual residents, including his/her personal contribution to the team function and individual ability to collaborate with various professionals.

Despite these unique assessment challenges, the pressure to rigorously measure collaboration among residents and their interprofessional teams is mounting. An increasing number of authorities cite team-based care as crucial to the practice of medicine and vital to health system reform [14–16]. Effective teamwork can enhance patient safety and improve healthcare quality [17, 18], and a common approach to assessment of interprofessional collaboration is critical to ensure that residents are competent in this essential milestone.

## Discussion

Given the importance of team-based care and the complexities of its assessment, residency educators need direction regarding best practices for assessing interprofessional collaboration milestones among their trainees. Residents and programs would benefit from consistency in assessments, especially since these data are used to make significant decisions about individuals and programs. Therefore, we propose common criteria for the assessment of interprofessional collaboration milestones that, in addition to the established standards for validity evidence [3, 4], include 4 components:

1. Assessment must measure competency of an individual resident
2. Assessment must be in the context of an interprofessional team (includes professions other than the resident’s profession)
3. Assessment must be ascertained via direct observation of the resident
4. Assessment must occur in a real-world clinical practice setting (such as a hospital ward, outpatient clinic, operating room, emergency room)

These criteria were derived from a comprehensive review of the literature regarding Graduate Medical Education (GME) milestones and interprofessional collaboration and teamwork assessment tools. Database search terms and strategy have been previously published [19], and were extended to encompass all specialties. The evidence-based rationale for each criterion is presented in the subsequent sections along with recommendations for existing assessment instruments that fulfill one or more of the criteria. Residency educators may choose to implement and/or adapt these instruments to assess interprofessional collaboration milestones.

### Interprofessional collaboration assessment of the individual resident

Milestone assessment necessitates attestation of individual resident competence. Unfortunately, among published instruments measuring interprofessional collaboration, the instruments with the most robust validity evidence measure collaboration of the team as a whole rather than at the individual level [6, 9, 10, 12, 13, 19–21]. Competencies for individual interprofessional collaboration have been proposed [16], but these competencies have not been translated into assessment tools. While the overall functioning of the team in clinical practice is of paramount importance, in residency training, milestone assessment requires measurement of the individual resident’s teamwork abilities and contributions to a team. The Ottawa Global Rating Scale [22–25] (OGRS) is one example of a published instrument that is well-suited for evaluation of an individual on a team. The OGRS is a 7-point descriptively-anchored scale of directly observed teamwork behaviors (problem solving, situational awareness, leadership, resource utilization, and communication) that has been validated among multilevel trainees from various specialties within simulated scenarios [22]. An important limitation of the OGRS is that it has not been studied among residents in real-world clinical practice settings. It also focuses on crisis resource management, so its applicability to individuals that are not in crisis mode is debatable. Validity studies of the OGRS within various inpatient and outpatient clinical settings are a necessary next step to optimize its use for milestone assessment.

### Collaboration assessment within an interprofessional team

Effective collaboration among professions enhances patient safety and healthcare quality [17, 18]. In GME, it is important to capture how well residents collaborate with individuals from multiple professions within the workplace. Tools such as the Nurse-Physician Collaboration Scale [26, 27], the ICU Nurse-Physician Questionnaire [28, 29], and the Jefferson Scale of Attitudes Toward Nurse-Physician Collaboration [30–32] have been used among residents to assess attitudes toward collaboration of the nurse-resident physician dyad, yet very few instruments measure resident collaboration with other health professionals such as pharmacists, therapists, social workers, clinical assistants, and administrative staff. Incorporating perspectives from the full spectrum of health professionals would significantly enrich milestone assessment data and provide a more complete picture of resident competence.

Residency educators interested in capturing viewpoints from multiple professions may consider utilizing the Team Climate Inventory (TCI) [11–13], which is a 44 to 65-item instrument that evaluates team participation, support, quality, discussion, clarity of objectives, and teamwork climate. Studies of TCI have included diverse non-physician professionals such as advanced practice providers, nurses, therapists, pharmacists, social workers, dieticians, clerical employees, and psychologists [11–13, 19]. Validity evidence for the TCI includes content validity, internal structure, relationships to other variables, and has been correlated with patient outcomes [12, 13, 20, 21]. The TCI does not, unfortunately, assess individual teamwork behaviors but rather examines teamwork climate as a whole in a self-reported manner. The Teamwork Mini-Clinical Evaluation Exercise (T-MEX) [33, 34] is a second instrument that incorporates observations from multiple health professionals, however it has only been studied within undergraduate medical education. Validity studies among residents are required to determine the utility of the T-MEX within GME.

### Interprofessional collaboration assessment via direct observation

Many evaluations of interprofessional collaboration rely upon resident opinion and self-assessment [35–37]. This type of subjective assessment, while valuable to obtain an overall picture of teamwork in many settings [9, 10, 12, 13, 20, 21], requires insight into team functioning and personal performance that some trainees may not possess. The ability to accurately self-assess varies among learners [38], and the extent to which self-assessment skills can be learned and improved is uncertain [39, 40]. To our knowledge, residents’ ability to accurately assess their own milestone performance has not yet been explored, but research among practicing physicians suggests that they may lack insight into their own ability to work in teams [41]. In addition, milestone assessments are designed to measure observable behaviors and skills. For all of these reasons, direct observation is preferable to self-assessment alone for assessing interprofessional collaboration.

Observations may be performed by supervising faculty, resident peers, nurses, pharmacists, allied health professionals, and others who interact with residents in the workplace. Direct observations can also be obtained from trained raters within simulated settings. Several simulation-based instruments have been developed within general surgery and surgical subspecialties. The Observational Teamwork Assessment for Surgery (OTAS) [42–46] measures communication and collaboration within the operating room using direct observation by raters trained to use this scale. Multiple studies have demonstrated validity evidence for the OTAS including content validity, response process, internal structure, relationships to other variables, and patient outcomes [42–49]. The Nontechnical Skills Evaluation Instrument (NOTECHS) [50–52] is similar to the OTAS in that it also assesses teamwork in the operating room using direct observation, but both are more commonly used for assessing teams or subteams rather than individuals. NOTECHS and OTAS have been simultaneously assessed and showed excellent agreement [50]. An additional tool, Non-Technical Skills for Surgeons (NOTSS), was initially validated in simulated operative settings and uses direct observation of individuals on a team [53]. NOTSS has substantial validity evidence and has helped inform the development of the SCORE (Surgical Council for Resident Education) modules for interprofessional education [54, 55]. Although the OTAS, NOTECHS and NOTSS were designed for use in the operating room, many of the content domains in these instruments, such as leadership, teamwork, communication, problem-solving, decision-making, and situation awareness, are applicable to numerous clinical environments including the hospital ward. Tools designed for use in operative simulation must be further adapted into diverse real-world practice settings to assure validity of these observations.

### Interprofessional collaboration assessment in a clinical practice setting

The primary purpose for assessing residents using milestones and other measures of competency is to ensure that, by the end of training, residents are ready for unsupervised practice. For this reason, it is essential that interprofessional collaboration is assessed within real-world clinical practice settings [56]. Many assessments of interprofessional collaboration utilize simulated scenarios [22, 36, 57], and while simulation is an excellent environment for residents to prepare for real clinical encounters, performance within simulation may not always mimic performance with a real patient. It is well known that learner behavior is influenced by environmental factors [58, 59] including workload, work intensity, stress and burn-out [60, 61]. As a result, residents who perform well in the controlled environment of an educational simulation may struggle when faced with the numerous challenges inherent in the practice milieu.

The Multi-disciplinary Team Performance Assessment Tool [62, 63] is an example of a direct observation assessment that has been applied among residents in a real-world clinical context, namely an inpatient oncology unit. This instrument focuses on team communication and captures each individual team member’s contributions to the cancer treatment team. Validity evidence includes content validity, response process, internal structure, and relationships to other variables. The Resident Leadership Scale assesses directly observed team leadership skills among internal medicine residents in the setting of an inpatient hospital ward [64]. Established validity evidence includes content, internal structure, and correlations with other measures of teamwork. Both the Multi-disciplinary Team Performance Assessment Tool and the Resident Leadership Scale have each been studied in a single clinical context (inpatient hospital unit), therefore future studies should explore the utility of these instruments in other clinical settings such as outpatient clinics and operating rooms.

## Summary

A common approach to the assessment of interprofessional collaboration milestones in GME is imperative. Effective teamwork is necessary to achieve safe, high quality patient care, and milestone evaluations inform crucial decisions within residency programs. Therefore, we suggest that measures of interprofessional collaboration milestones use direct observation of an individual resident on an interprofessional team in a real-world clinical practice setting. While many existing instruments meet one or more of these criteria, these instruments have significant limitations. Future studies should focus on adapting current assessments to more fully encompass these important components within graduate medical education clinical environments.
